# Analysis of the *Pythium ultimum *transcriptome using Sanger and Pyrosequencing approaches

**DOI:** 10.1186/1471-2164-9-542

**Published:** 2008-11-15

**Authors:** Foo Cheung, Joe Win, Jillian M Lang, John Hamilton, Hue Vuong, Jan E Leach, Sophien Kamoun, C André Lévesque, Ned Tisserat, C Robin Buell

**Affiliations:** 1 The J. Craig Venter Institute, 9704 Medical Center Dr, Rockville, MD 20850 USA; 2 The Sainsbury Laboratory, Colney Lane, Norwich, NR4 7UH, UK; 3 Colorado State University, Department of Bioagricultural Sciences and Pest Management, C129 Plant Sciences, Ft. Collins CO 80523, USA; 4 Michigan State University, Department of Plant Biology, East Lansing MI 48824, USA; 5 Agriculture and Agri-Food Canada, Ottawa, ON, K1A 0C6, Canada

## Abstract

**Background:**

*Pythium *species are an agriculturally important genus of plant pathogens, yet are not understood well at the molecular, genetic, or genomic level. They are closely related to other oomycete plant pathogens such as *Phytophthora *species and are ubiquitous in their geographic distribution and host rage. To gain a better understanding of its gene complement, we generated Expressed Sequence Tags (ESTs) from the transcriptome of *Pythium ultimum *DAOM BR144 (= ATCC 200006 = CBS 805.95) using two high throughput sequencing methods, Sanger-based chain termination sequencing and pyrosequencing-based sequencing-by-synthesis.

**Results:**

A single half-plate pyrosequencing (454 FLX) run on adapter-ligated cDNA from a normalized cDNA population generated 90,664 reads with an average read length of 190 nucleotides following cleaning and removal of sequences shorter than 100 base pairs. After clustering and assembly, a total of 35,507 unique sequences were generated. In parallel, 9,578 reads were generated from a library constructed from the same normalized cDNA population using dideoxy chain termination Sanger sequencing, which upon clustering and assembly generated 4,689 unique sequences. A hybrid assembly of both Sanger- and pyrosequencing-derived ESTs resulted in 34,495 unique sequences with 1,110 sequences (3.2%) that were solely derived from Sanger sequencing alone. A high degree of similarity was seen between *P. ultimum *sequences and other sequenced plant pathogenic oomycetes with 91% of the hybrid assembly derived sequences > 500 bp having similarity to sequences from plant pathogenic *Phytophthora *species. An analysis of Gene Ontology assignments revealed a similar representation of molecular function ontologies in the hybrid assembly in comparison to the predicted proteomes of three *Phytophthora *species, suggesting a broad representation of the *P. ultimum *transcriptome was present in the normalized cDNA population. *P. ultimum *sequences with similarity to oomycete RXLR and Crinkler effectors, Kazal-like and cystatin-like protease inhibitors, and elicitins were identified. Sequences with similarity to thiamine biosynthesis enzymes that are lacking in the genome sequences of three *Phytophthora *species and one downy mildew were identified and could serve as useful phylogenetic markers. Furthermore, we identified 179 candidate simple sequence repeats that can be used for genotyping strains of *P. ultimum*.

**Conclusion:**

Through these two technologies, we were able to generate a robust set (~10 Mb) of transcribed sequences for *P. ultimum*. We were able to identify known sequences present in oomycetes as well as identify novel sequences. An ample number of candidate polymorphic markers were identified in the dataset providing resources for phylogenetic and diagnostic marker development for this species. On a technical level, in spite of the depth possible with 454 FLX platform, the Sanger and pyro-based sequencing methodologies were complementary as each method generated sequences unique to each platform.

## Background

*Pythium *species are ubiquitous fungal-like organisms in the Kingdom Straminipila. They are related to the oomycete plant pathogens *Phytophthora *and downy mildews (e.g. *Bremia, Peronospora*, and *Plasmopora*) in the Peronosporales and to *Saprolegnia *and *Aphanomyces *in the Saprolegniales. They differ from the true fungi (Eumycota) in that they are diploid, have coenocytic hyphae containing β-1–3 glucans and cellulose in their cell walls, reproduce sexually by fertilization of oogonia by antheridia and, in many species, form motile, biflagellate zoospores. Nevertheless, *Pythium *species have many similarities to true fungi in the way they obtain nutrients from the environment or attack plants [1].

The genus *Pythium *is biologically and ecologically diverse. Although approximately 250 *Pythium *species have been described, only half that number has been recognized as valid descriptions [2-4]. *Pythium *species have been divided into 11 phylogenetic clades based on rDNA ITS and other sequence data [3]. Most are soil inhabitants although some reside in salt water estuaries and other aquatic environments. Most *Pythium *species are saprophytic or facultative plant pathogens [4]. They are among the most important plant pathogens and cause a variety of diseases including seed rots and damping-off, root, stem and fruit rots, foliar blights and postharvest decay [5-8]. A few non-phytopathogenic species show promise as biological control agents [9]. Other *Pythium *species are parasites of insects [10] and fish [4] and at least one species (*P. insidiosum*) can infect animals and causes skin and bone lesions in pigs, dogs, and humans [11].

*Pythium ultimum *is a cosmopolitan plant pathogen with a broad host range. It is one of the most pathogenic *Pythium *species on corn, soybean, wheat, ornamentals, and many other crops [9]. It is homothallic, and in most cases, self fertile, although some outcrossing may occur [12]. *P. ultimum *is actually a species complex with three main morphological types. *P. ultimum *var. *ultimum*, the most common type, produces oospores but very rarely sporangia and zoospores, and then only at cool temperatures [4]. It is part of a large uniform clade based on rDNA ITS sequence comparisons which includes the neotype strain [3] as well as the isolate used to generate the Expressed Sequence Tags (ESTs) in this study. *P. ultimum *var. *sporangiiferum *produces oospores and sporangia at room temperature [4]. In Barr et al. [13], the four *P. ultimum *var. *sporangiiferum *isolates formed a unique genotype (U6) based on isozyme analysis whereas in Francis et al. [12] the two isolates of this variety were in two distinct genotypes, one belonging to the most common genotype of *P. ultimum *var. *ultimum *strains. It is unclear at this point whether or not the more infrequent but unique genotypes of *P. ultimum *such as the genotype U6 should be split into different species, reducing the observed genetic diversity within this species. Having more information on Single Nucleotide Polymorphisms would help to delineate gene flow and species boundaries in this ubiquitous species complex. Isolates in the third morphological type do not produce oospores in culture but can be identified based on other morphological characteristics, molecular studies, and the fact that they can be crossed with oospore producing strains of *P. ultimum *[3,12-14].

ESTs are a robust method for gene discovery and for identifying transcripts involved in specific biological processes. ESTs have been developed for over 40 oomycete and fungal pathogens [15]. For the oomycetes, EST projects have provided not only the first insight into the gene complement for these plant pathogens but also sets of genes involved in a number of biological processes [16-24]. With the advent of reduced costs and higher throughput sequencing methods, ESTs can be economically generated for a wider range of organisms and a greater breadth of conditions, thereby providing a more comprehensive assessment of an organism's transcriptome. One emerging platform for *de novo *sequencing of transcriptomes or genomes is pyrosequencing [25]. However, the pyrosequencing-derived read lengths on the GS20 and FLX platform are substantially shorter than conventional Sanger-based sequencing which is problematic for assembly into a non-redundant set of consensus sequences. This limitation can be addressed through increased sequencing coverage of the transcriptome and/or through hybrid approaches in which conventional Sanger sequences are co-assembled [26] with pyrosequencing-derived sequences. Thus, for *de novo *sequencing of a transcriptome, both approaches are advantageous with pyrosequencing providing a deep representation of the transcriptome and Sanger-derived sequences providing seed sequences for assembly purposes. In this study, we report the first set of ESTs for this agriculturally important plant pathogen and provide a direct comparison of gene discovery that can be obtained with two high throughput sequencing methods, Sanger-based chain termination and 454 FLX pyrosequencing.

## Results and Discussion

### Sanger-based sequencing of a normalized cDNA library

**Figure 1:**
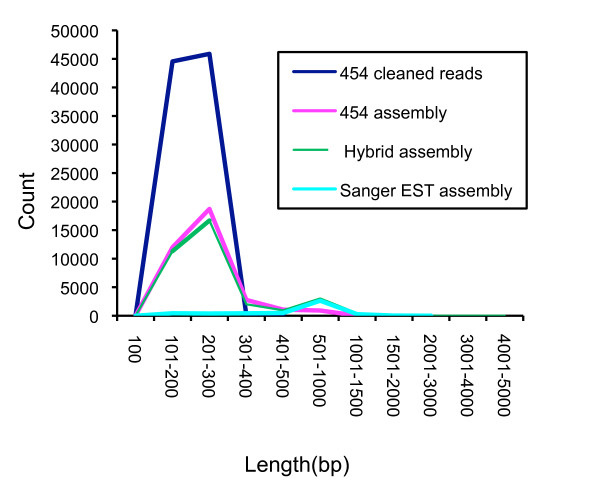
**Size distribution of assemblies and reads**. Length distribution (in base pairs) of reads and assemblies from the Sanger, 454, and hybrid assemblies.

**Figure 2:**
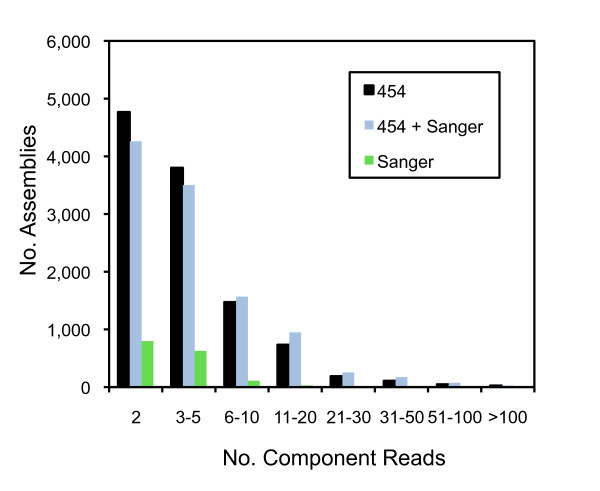
**Component size distribution.** The number of reads in each assembly is plotted for each of the three assemblies (Sanger reads, 454 reads, and hybrid assembly).

A normalized cDNA library was generated from RNA isolated from hyphae growing on two contrasting media conditions, nutrient-rich and nutrient-starved conditions. A total of 9,578 reads from Sanger-based sequencing was generated; these assembled into 4,689 unique sequences (assemblies plus singletons) with an average length of 596 nucleotides and a total length of 2.8 Mb (Table 1, Figure 1). The majority of assemblies contain two component reads while very few assemblies contain six or more component reads (Figure 2). Overall, clustering and assembly of the reads resulted in a two-fold reduction in the number of unique sequences with few EST components per assembly consistent with an effective normalization treatment of the cDNA prior to library construction. We confirmed the origin of the ESTs by aligning the assemblies to a draft *P. ultimum *BR144 genome sequence [27]. For the Sanger-only assembly, 4,127 (88%) sequences (1,408 assemblies, 2,719 singletons) aligned to the draft genome. Of the 4,689 unique sequences, 2,345 (50.0%) had sequence similarity with an entry in the UniRef100 database [28]. Within the set of 4,689 unique sequences (assemblies and singletons), 4,444 open reading frames (ORFs) could be predicted. Of these, 3,676 ORFs contained an ATG translational start codon while 2,413 had both an ATG start codon and a stop codon. Of these 2,413 ORFs, 1,421 (58.6%) have sequence similarity with a protein sequence in the UniRef100 database that contains the corresponding start codon and are thus potentially full-length. The length of top UniRef100 matches ranged between 50 to >500 amino acids in length, indicating that the sequences and therefore the cDNAs generated from this library were not biased towards either long or short sequences.

**Table 1:** Sequence and assembly statistics

	**Pyrosequencing ESTs**	**Sanger ESTs**	**Pyrosequencing + Sanger ESTs**	**Pyrosequencing assembly**	**Sanger assembly**	**Hybrid Assembly**
No. sequences	90,664	9,578	90,664 + 9,578 = 100,242	35,507 (11,155 assemblies)	4,689 (1,584 assemblies)	34,495 (10,811 assemblies)
Total bases (bp)	17,315,041	4,811,851	22,126,892	8,274,073	2,796,219	9,552,701
Minimum length (bp)	100	100	100	100	100	100
Average length (bp)	190	502	220	233	596	276
Maximum length (bp)	425	1,002	1,002	1,385	4,283	4,471
%GC	50.3	51.1	50.5	51.3	53.3	51.7
%N	0.04	0	0.03	0.05	0	0.04

### 454 Pyrosequencing sequencing and assembly

A single half-plate pyrosequencing run on the same normalized cDNA used in the Sanger-based sequencing generated 139,839 reads, which after trimming and cleaning, yielded 90,664 usable reads (>100 base pairs) with an average length of 190 nucleotides and a total length of 17.3 Mb (Table 1). After clustering and assembly using the TGICL clustering utilities [29], 66,312 sequence reads were incorporated into 11,155 assemblies and 24,352 singletons for a total of 35,507 unique sequences. Assemblies had an average length of 233 bp with a maximum length of 1,385 bp (Table 1, Figure 1). For the 454 assembly, 25,285 (71%) sequences (9,226 assemblies, 16,059 singletons) aligned to the draft *P. ultimum *BR144 genome sequence. Of these 35,507 unique sequences, 7,991 (22.5%) had a protein match with UniRef100 [28], significantly less than what was observed for the Sanger-derived ESTs. If only sequences > 500 bp were considered, 71% had a match with an entry in UniRef100 suggesting that the short length of the 454 reads impact the ability to detect similar sequences. As the pyrosequencing library preparation involves random shearing of the normalized (but uncloned) cDNA population [25], the pyrosequencing reads originate from random locations within each cDNA and may have either orientation. The majority of assemblies contain 2–5 reads, while in contrast only a few of the assemblies contained over 100 pyrosequencing EST reads (Figure 1). This is presumably due to both the length of the individual reads, the normalization of the cDNA population, and the coverage of the transcriptome represented in this dataset.

### Hybrid assembly of Sanger and Pyrosequencing-based reads

A hybrid assembly was constructed using the pyrosequencing- and Sanger-based EST reads which were clustered and assembled using the TGICL clustering utilities [29]; of these, 10,811 were assemblies and 23,684 were singletons for a total of 34,495 unique sequences (Table 1). Since the reads used in the hybrid assembly were predominantly derived from the pyrosequencing dataset, with the exception of the maximum assembly length, the assembly length and EST component statistics are similar to that for the pyrosequencing-only reads (Figure 1). In the hybrid assembly, a majority of assemblies are short with an average length of 276 base pairs (maximum length of 4,471 base pairs (Table 1)) and contain few component reads (Figure 2). The majority of assemblies contain 2–5 EST reads while only a limited number of assemblies contain over 100 EST reads. For the hybrid assembly, 24,140 (70%) sequences (8,922 assemblies, 15,218 singletons) aligned to the draft *P. ultimum *BR144 genome sequence. Within the hybrid assembly, 8,618 had a protein match using UniRef100 [28] significantly less than what was observed for the Sanger-derived ESTs yet consistent with the abundance of 454 reads in the overall hybrid assembly. If only sequences > 500 bp within the hybrid assembly were considered, 69% had a match with an entry in UniRef100. Pyrosequencing using the 454 platform is reported to have reduced accuracy in homopolymer regions [25]. Examination of homopolymer regions (>4 nucleotides) detected between the Sanger- and the 454-derived reads revealed differences in homopolymer length between the Sanger and 454-reads in 837 instances, primarily in A or T homopolymer regions (407 A, 323 T, 65 G, 42 C).

A total of 1,110 unique sequences in the hybrid assembly were derived from Sanger sequencing alone. Although the majority of these had no similarity with entries in the UniRef100 database (Additional Data Files 1 and 2), 907 (81.7%) of them mapped to the *Pythium *genome at very high stringencies (95% identity, 95% coverage with a minimum of 100 bp of the Sanger read; Cheung and Buell, unpubl.), suggesting that these are not contaminants or artifacts within the cDNA library. Of the 1,110 unique sequences derived solely from Sanger reads, 576 had no match even at a very low threshold (BLASTN E-10 [30] or BLAT [31], default settings) with the pyrosequencing-derived sequences. Further analysis of these 576 Sanger-only reads showed that 461 (80%) could be mapped to the *Pythium *genome with high stringency (≥ 95% identity, ≥ 95% coverage, of a minimum of 100 bp of the Sanger read). An analysis of average read length revealed that these Sanger sequences were slightly shorter on average (99 bp) than the complete set of Sanger sequences which would reduce to the probability of co-assembling with 454 reads during the hybrid assembly process (average length of 497 nucleotides in Sanger-only singletons and assemblies vs 596 nucleotides in the complete set of Sanger singletons and assemblies). Although the GC content did not differ substantially between the Sanger-only sequence set and the complete Sanger sequence set (51% vs 53%), other sources of possible bias could be the cloning and replication in *E. coli *which may amplify select sequences in the library compared to the 454 sequences. These results suggest that a small proportion of ESTs generated by Sanger sequencing are not present in the dataset generated by pyrosequencing.

### Biological features of the *P. ultimum* transcriptome

The top 50 assemblies containing the most ESTs from all three builds (pyrosequencing reads only, Sanger reads only, hybrid assembly) ranged from 10 to 2,654 ESTs (Additional Data Files 3, 4, 5). Most of the protein matches to the top 50 assemblies from all three builds were to *Phytophthora *species. In general, the most common annotations based on similarity to UniRef100 entries were housekeeping genes although 17 of the top 50 deepest assemblies from the hybrid assembly had no match to an entry in UniRef100 (Additional Data File 3).

To determine how similar the *P. ultimum *transcriptome is to other plant pathogenic oomycetes, we compared our EST sequences with the predicted proteomes and transcriptomes available for four *Phytophthora *species: *P. infestans *(transcriptome and predicted proteome), *P. parasitica *(transcriptome), *P. ramorum *(predicted proteome), and *P. sojae *(transcriptome and predicted proteome) [32,33]. Using BLASTX with the predicted proteome data sets, 16,139 (46.8%) of the *P. ultimum *hybrid assembly sequences matched a sequence present within the *P. infestans*, *P. ramorum*, or *P. sojae *predicted proteomes. Using TBLASTX with a cutoff criterion of E = 1e-10, 14,021 (40.6%) of the *P. ultimum *hybrid sequences matched a sequence within the three *Phytophthora *transcriptomes with only 945 (2.7%) of the *P. ultimum *sequences that align with a predicted *Phytophthora *protein not matching a *Phytophthora *transcript. Using BLASTX with a cutoff criterion of E = 1e-5 against the UniRef100 database, 8,618 (25.0%) of the *P. ultimum *hybrid sequences matched the UniRef database, of which 673 (2.0%) did not also match a *Phytophthora *transcript or predicted protein. Collectively, from all three datasets (*Phytophthora *spp. predicted proteomes, *Phytophthora *spp. transcriptomes, and the UniRef database), 16,738 (48.5%) of the *P. ultimum *hybrid sequences do not have a match. This is most likely attributed to the short nature of the majority of the hybrid sequences. Indeed, if only sequences ≥ 500 bp within the hybrid assembly are considered (3,515 total sequences), the percentage of sequences with an alignment to the three data types significantly increases; 3,201 (91.1%) align to the *Phytophthora *spp. predicted proteomes, 2,959 (84.2%) align to the *Phytophthora *spp. transcriptomes, and 2,427 (69%) align to the UniRef database, with only 252 (7.2%) without a match to any sequence within these data sets.

**Figure 3:**
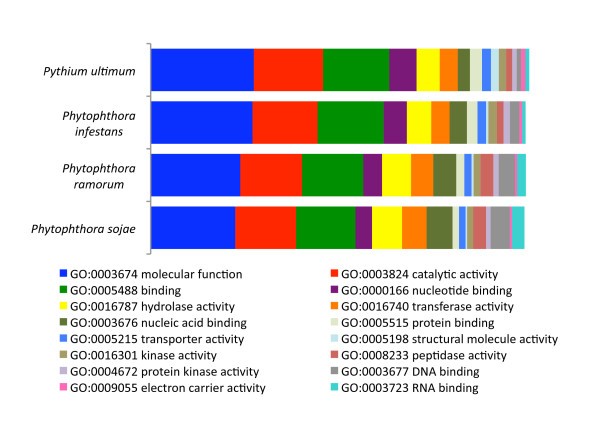
**Distribution of GOSlim assignments in the *P. ultimum* hybrid EST sequences compared to the predicted proteomes of three *Phytophthora* species.** The percentage of assignments of the total GOSlim assignments are shown. Only GOSlim terms present at level of 1% or greater in any of the four species are shown.

We also performed a comparative analysis of Gene Ontologies [34] with *P. ultimum *and the three *Phytophthora *proteomes. As shown in Figure 3, the normalized cDNA obtained from hyphae grown in rich and nutrient-starved conditions encoded a broad set of transcripts represented within the molecular function gene ontologies. Furthermore, the representation is very similar to that of the complete proteomes of the three plant pathogenic *Phytophthora *species suggesting that our approach of cDNA normalization, coupled with deep sequencing, provided a near complete representation of the *P. ultimum *transcriptome.

It recently emerged that *Phytophthora *and downy mildew species secrete a vast repertoire of effector proteins that modulate host defenses and enable pathogenicity [35,36]. The extent to which *Pythium *species also rely on secreted effectors to colonize host tissue is unclear. We therefore examined the *P. ultimum *ESTs for similarity to known oomycete effectors. We first scanned the *P. ultimum *EST hybrid assembly for candidate cytoplasmic effectors of the RXLR and Crinkler (CRN) families [36,37] using a combination of the PexFinder algorithm [23] to identify putative secreted protein genes and sequence similarity searches. An hmm profile based on an alignment of known RXLR-EER effectors [38] revealed one *P. ultimum *assembly (asmbl_7845) as a putative positive. *P. ultimum *asmbl_7845 encodes an ORF with a signal peptide (SignalP HMM score = 0.921) followed by the RLLRSAGDVESSAVDDAAR sequence with similarity to the RXLR-DEER motif (Additional Data File 6A–B). The identification of only a single putative RXLR effector is surprising and contrasts to the common occurrence of RXLR effectors in similar sets of *Phytophthora *ESTs [21]. Apparently, RXLR effectors are not as widely present or expressed in *P. ultimum *as noted for *Phytophthora *species. There are several possible explanations. It is possible that *P. ultimum *does not have RXLR effector genes or has a highly reduced set compared to *Phytophthora*. This would be consistent with observations [39] that suggested that RXLR effectors are delivered through haustoria, specialized infection structures that are not produced by *Pythium*. The RXLR motif is similar in sequence, position and function to the *Plasmodium *Pexel/Host translocation motif [40]. The possible absence of RXLR effectors in *P. ultimum *indicates that although the motif is conserved across divergent parasitic eukaryotes it may not be ubiquitous in oomycetes. Four Crinkler-like sequences were identified among the *P. ultimum *hybrid assembly. In this case, the similarity to *Phytophthora *Crinklers was more convincing than for the single RXLR effector candidate with BLASTX E values as low as e-48. Clearly, these sequences displayed the consensus LXLYLAXR instead of the LXLFLAK motif that defines canonical *Phytophthora *and downy mildew Crinklers [37,38] (Additional Data File 6C). In summary, we detected one potential candidate RXLR and several Crinkler effectors in *P. ultimum*, however, they are not as abundantly represented among the examined *P. ultimum *ESTs as they are in *Phytophthora *ESTs from similar developmental stages [21].

We also searched the *P. ultimum *assemblies for similarities to other oomycete effectors. We detected three assemblies with similarity to oomycete Kazal-like serine protease inhibitors [41,42] and another three with similarity to cystatin-like protease inhibitors [43] (Additional Data File 7). In *Phytophthora*, these apoplastic effectors are known to inhibit defense related proteases of plants [41-43]. In addition, at least 13 assemblies with similarity to elicitins were identified (Additional Data File 7). Elicitins are secreted lipid-binding oomycete proteins that trigger defense responses in plants [36]. These elicitins showed significant similarity to previously described *Phytophthora *and *Pythium *elicitins with their characteristic cysteine-rich domain [44,45]. Six assemblies were most similar to sylvaticin, a secreted elicitin of *Pythium sylvaticum *of unknown function [45]. The same assemblies showed significant similarity with the elicitin-like protein of *Pythium oligandrum *[46] but the homology was much lower than that of *P. sylvaticum *(E value e-11). This is consistent with the taxonomy and phylogeny of these species. Both *P. ultimum *(clade I) and *P. sylvaticum *(clade F) belong to the globose sporangia group of *Pythium *whereas *P. oligandrum *(clade D) belongs to a different group with contiguous sporangia [47].

Unlike *Phytophthora *spp., *Pythium *and other oomycetes like *Saprolegnia *spp. are not thiamine auxotrophs. Torto et al. [48] reported sequences with similarity to thiamine biosynthesis enzymes among ESTs of the fish pathogen *Saprolegnia parasitica *that are missing in the genome sequences of *P. sojae*, *P. ramorum*, *P. infestans*, and *Hyaloperonospora parasitica*. We identified four *P. ultimum *sequences within the hybrid assembly (asmbl_312; asmbl_353; asmbl_1697, and 334590_1440_2761) with similarity to the *S. parasitica *thiamine biosynthesis enzyme. This finding is consistent with the knowledge that *P. ultimum *can synthesize thiamine. The thiamine biosynthesis gene was apparently lost during the evolution of the *Phytophthora*/downy mildews lineage and could serve as a potential phylogenetic marker among Saprolegniales and the genus *Pythium*.

Oomycetes are often characterized by the ultrastructure of their flagellar apparatus. Although the strain we sequenced and *P. ultimum *var. *ultimum *are generally not known to produce zoospores *in vitro*, it might be possible that some flagellar associated proteins are expressed as was observed in *Phytophthora *grown under conditions that do not produce zoospores [21]. Some of the flagellar associated proteins from *Chlamydomonas reinhardtii *(177 in total, Additional Data File 8) that we identified in our ESTs are predicted to be commonly expressed in other structures/tissues than zoospores (e.g. calmodulin or elongation factor). As shown by Randall *et al*[21], we also found evidence of expression of dynein related to the flagellar apparatus (E values between e-6 and e-38) but we also identified several flagellar basal body proteins (E values between e-8 and e-21) that were expressed.

Random candidate markers for population genetic studies can also be derived from simple sequence repeats (SSRs). Within the hybrid assembly, a total of 179 SSRs were identified within 164 sequences. Among the SSRs, monucleotides (45) were the most abundant followed by dinucleotides (44), trinucleotides (33), pentanucleotides (27), tetranucleotides (26), and hexanucleotides (4) (Additional Data File 9). Lee and Moorman [49] developed SSR markers for *Pythium aphanidermatum*, *P. cryptoirregulare *and *P. irregulare *from an SSR enriched library. Primers P18CCA1-41, P18TTC1-42, P18CAT1-74 amplified SSRs in *P. ultimum *but were not found in this EST library. It is possible that the genes in which these SSRs were located were not expressed, that these SSRs were in non-coding regions, or that the primers designed from other species worked on *P. ultimum *even if they had mismatches.

## Conclusion

In this study, we report the integration of data from Sanger-based chain termination and 454 FLX pyrosequencing technologies. The two technologies were highly complementary although the shorter read length in the pyrosequencing-derived ESTs is problematic in that they limit biological interpretations due to the reduced information content in the predicted protein sequence. Thus, while the pyrosequencing did provide depth of coverage, we were able to generate a more robust set of transcribed sequences for *Pythium *by co-assembly of Sanger and pyrosequencing derived ESTs. Furthermore, even with a greater depth of ESTs provided through the 454 FLX platform, the two sequencing methodologies generated sequences unique to each platform. The results presented contain an ample number of candidate polymorphic markers providing resources for potential phylogenetic and diagnostic and strain marker development for this agriculturally important group of plant pathogens. We expect that a similar analysis using other species and integration of data from 454 FLX pyrosequencing technologies would work synergistically with existing or new EST data and identify new genes/transcripts at a very cost effective and efficient manner.

## Methods

### Materials and Organismal Methods

Yeast extract broth (30 g/L sucrose, 1 g/L KH_2_PO_4_, 0.5 g/L MgSO_4 _7H_2_O, 0.5 g/L KCl, 10 mg/L FeSO_4 _7H_2_O, 1 g/L yeast extract) was inoculated with several crushed plugs of 3 day old *P. ultimum *strain DAOM BR144 (= ATCC 200006 = CBS 805.95) grown on 10% V8 agar and incubated at room temperature on a shaker (Lab-Line Instruments, Melrose Park, IL) at 200 rpm for 2 days. Plugs of the same isolate were covered with modified Plich medium (Kamoun, S., unpub.) in a Petri dish and incubated in the dark at room temperature for 10 days. Mycelia from both media were rinsed with 50 ml of sterile water and harvested by straining through sterile cheesecloth, weighed and then ground in liquid nitrogen using a mortar and pestle.

### Molecular and Bioinformatics Methods

RNA was extracted from this tissue using TRIzol reagent (Invitrogen, Carlsbad, CA) according to the manufacturer's instructions. Equal masses of total RNA from the two growth conditions were used for construction of a normalized cDNA population and subsequently, a normalized cDNA library. Using the services of Evrogen [50], full length cDNA was constructed using the Smart cDNA cloning methodology [51] and normalized using a duplex-specific nuclease [50]. Two methods were used to generate sequences from the normalized cDNA: Sanger-based dideoxy chain termination and Roche 454-FLX pyrosequencing [25]. Sanger-based sequences were generated from end sequencing of cDNA clones using standard high throughput sequencing methods on an ABI 3730xl sequencer. Sequences were trimmed to remove vector, low-quality, and *E. coli *sequences using Lucy [52] and iterative runs of the TIGR Seqclean Tool [53]. Accessions are available in GenBank dbEST (EL774305–EL783216 and ES607153–ES608087). Pyrosequencing was performed on a Roche 454 FLX sequencer [25] using a half-plate. Pyrosequencing reads were trimmed to remove adaptors using TIGR Seqclean Tool [14]. Short sequences (< 100 nucleotides) were removed from the build process as the addition of extremely short sequences will lead to false joining of ESTs, chimeras that were sequenced, and reduced quality of unique sequences. Sequences were deposited in GenBank dbEST under accession numbers FE969956–FF060620.

Pyrosequencing and Sanger cDNA reads were assembled using the TGICL package [29] as was the hybrid assembly using the default parameters as described in Childs et al. [54] with the exception that sequences < 100 nucleotides were excluded. Sequences were searched against the UniRef100 database (Release 12.0) using BLASTX with a cutoff criterion of E = 1e-5. The EST assemblies were aligned with a draft assembly of the *P. ultimum *BR144 genome [27] using BLAT [31]. *Pythium *EST assemblies were downloaded from The Comprehensive Phytopathogen Resource Trancript Assemblies website [15] and *Phytophthora *genome sequences were downloaded from GenBank. Transcriptomes of *P. ultimum *version 1.0, *P*. *infestans *version 3.0, *P. sojae *version 1.0, and *P. parasitica *version 2.0 were aligned using TBLASTX with cutoff criteria of E = 1e-5. *P. ultimum *ESTs were aligned with the *P. ultimum *[55], *P*. *infestans *[56], *P. ramorum *[33] and *P. sojae *[33] genomes with TBLASTX requiring a minimium of 40% length matching. For the Gene ontology associations, the three *Phytophthora *proteomes were searched against the current UniRef100 database using BLASTP and a cutoff of 1e-5. GO ids were assigned using the EBI Uniprot Knowledgebase GOA file [57] using the top UniRef100 hit. The assigned GO identifiers were then mapped to the Generic GOSlim ontology using the map2slim tool [58]. Using BLASTX and a cutoff of 1e-5, the hybrid EST set was searched against 892 *Chlamydomonas reinhardtii *proteins flagged in GenBank as being associated with the flagellar apparatus to identify transcripts associated with the flagellar mechanism in *Pythium*. Perfect mononucleotide to hexanucleotide simple sequence repeats were identified using MISA [59]. Perl scripts were used to specify the minimum number of the following repeats for microsatellites (unit size/minimum number of repeats): (1/20) (2/10) (3/7) (4/5) (5/5) (6/5).

## Authors' contributions

FC was responsible for analysis of the EST data. JML was responsible for isolating mRNA and data analysis. JW and SK were responsible for analysis of effectors. NT, JEL, and CAL were responsible for strain selection, experimental design, data analysis, and discussion. JH was responsible for data analysis. CRB was responsible for experimental design, sequencing, data analysis, and discussion.

## Supplementary Material

Additional file 1 **Hybrid assembly singleton reads derived solely from Sanger reads.** Click here for file

Additional file 2 **Hybrid assemblies derived solely from Sanger reads.** Click here for file

Additional file 3 **Top 50 largest assemblies from the hybrid assembly.** Click here for file

Additional file 4 **Top 50 largest assemblies from 454-pyrosequencing only assembly.** Click here for file

Additional file 5 **Top 50 largest assemblies from Sanger only sequence assembly.** Click here for file

Additional file 6 **Alignments of *P. ultimum *sequences to effectors and Crinkler proteins.** Click here for file

Additional file 7 **Assemblies with similarity to protease inhibitors and elicitins.** Click here for file

Additional file 8 ***Chlamydomonas*****flagellar proteins identified in the hybrid EST assembly.** Click here for file

Additional file 9 **Simple Sequence Repeats identified in the hybrid assembly.** Click here for file
