# Supplementary material for: Analysis of the Pythium ultimum transcriptome using Sanger and Pyrosequencing approaches
Source: BMC Genomics. 2008 Nov 15;9:542. doi: 10.1186/1471-2164-9-542 (PMC2612028; doi:10.1186/1471-2164-9-542)
Supplement: Additional file 6 — Alignments of P. ultimum sequences to effectors and Crinkler proteins. [file 1471-2164-9-542-S6.pdf]

Additional Data File 6. Alignments of *P. ultimum* sequences to effectors and Crinkler proteins.

A-B. *P. ultimum* asmbl_7845 encodes a putative secreted protein with similarity to RXLR –DEER effectors. (A) Alignment of asmbl_7845 to a consensus sequence of the RXLR-DEER motif (Win et al. 2007, Plant Cell 19:2349). **(B)** Predicted amino acid sequence of asmbl_7845 highlighting the signal peptide (blue) and RXLR-ERR motif (red). Note that the C-terminal sequence is probably missing from the ESTs.

A

```
Win et al hmm                dvsgkRfLRahetsaaeeddeddEERgltkt
                             +vs++R+LR++  +    +  dd  Rg + +
asmbl_7845          48       NVSATRLLRSAGDVESSAV--DDAARGYSPP    76
```

B

```
>asmbl_7845_F2 - Signal peptide HMM score = 0.921 - length = 28
MIPKPNSIRHTISKRSLLFSFFGASASACTSKLPRIDAVSEPTPWSENVSATRLLRSAGDVESSAVDDAARGYSPPI
```

C. *P. ultimum* assemblies with similarity to the Crinkler family. (C) Best blastx hits in GenBank nonredundant database. Alignment of the *P. ultimum* sequences to a consensus sequence of the Crinkler LXLFLAK motif (Win et al. 2007, Plant Cell 19:2349). **(D)** Alignment of the *P. ultimum* sequences to a consensus sequence of the Crinkler LXLFLAK motif (Win et al. 2007, Plant Cell 19:2349).

C

Identifier	Best blastx hit	E value
asmbl_1776	CRN-like CRN3 [*Phytophthora infestans*]	5e-14
asmbl_5037	CRN-like CRN3 [*Phytophthora infestans*]	3e-07
asmbl_8973	CRN-like CRN10 [*Phytophthora infestans*]	2e-48
PUNBR20TV	crinkling and necrosis-inducing protein CRN1 [*Phytophthora infestans*]	0.001

D

LQLFLAKK	*Phytophthora* consensus
LLLYLAKR	asmbl_1776
LKLYLASR	asmbl_5037
LTLYLARK	asmbl_8973
LELYLAIK	PUNBR20TV
LxLYLAxR	*Pythium ultimum* consensus
